# Pathogen-Induced Lysosomal Membrane Permeabilization: A Critical Interface Between Host Defense and Cell Death

**DOI:** 10.3390/ijms27031515

**Published:** 2026-02-03

**Authors:** Xiao Liu, Zhan Li, Yuru Hu, Tao Li, Hui Wang

**Affiliations:** 1 State Key Laboratory of Pathogen and Biosecurity, Academy of Military Medical Sciences, Beijing 100071, China; liuxiao0279@163.com (X.L.); yexi19881214@126.com (Z.L.); huyururu@126.com (Y.H.); 2 School of Basic Medical Sciences, Anhui Medical University, Hefei 230032, China

**Keywords:** lysosome, lysosomal membrane permeabilization, pathogen infection, cell death, cathepsin

## Abstract

During pathogen infection, lysosomes are not only pivotal targets exploited by pathogens to evade host defenses and induce cell death, but also an essential frontline of host protection that restricts infection by degrading invading microbes and repairing membrane damage. A broad spectrum of pathogens—including bacteria, viruses, protozoa, and fungi—can trigger lysosomal membrane permeabilization (LMP), resulting in the leakage of lysosomal contents into the cytosol. The released lysosomal factors can selectively activate distinct cell-death programs, including apoptosis, pyroptosis, ferroptosis, and necroptosis. These cell-death processes may limit pathogen dissemination by eliminating infected cells, yet they can also exacerbate disease through excessive inflammatory responses and tissue injury. In this review, we highlight recent advances and systematically discuss the determinants of lysosomal membrane stability, methods for detecting LMP, and LMP-driven cell-death modalities, and we summarize the mechanisms and consequences of pathogen-induced LMP.

## 1. Introduction

Lysosomes are vesicular organelles enclosed by a single biological membrane and enriched in a variety of acidic hydrolases [1]. Their physiological functions include the degradation of extracellular pathogens and other foreign materials internalized through endocytosis or phagocytosis, as well as the turnover and recycling of intracellular components—such as aged organelles and aberrant proteins—via autophagy [2]. Shortly after the discovery of lysosomes, the biologist Christian de Duve proposed the “suicide bag” hypothesis [3], which postulated that lysosomes, as membrane-bound reservoirs of acidic hydrolases, may undergo membrane rupture in response to cellular damage, aging, or external stimuli. The consequent release of lysosomal hydrolases into the cytosol would then trigger the degradation of cellular constituents, ultimately leading to cell death. This process, characterized by the loss of lysosomal membrane integrity and the leakage of lysosomal contents, was later formally defined as lysosomal membrane permeabilization (LMP) [4]. During pathogen infection, LMP exhibits a pronounced “double-edged sword” nature. On the one hand, LMP can support host defense by translating lysosomal damage into programmed cell death and amplified immune responses, thereby promoting pathogen clearance. On the other hand, uncontrolled cell death may result in dysregulated immune responses, cytokine storms, and tissue damage [5]. In this context, some intracellular pathogens may exploit LMP to exit infected cells and enhance dissemination. Following LMP, lysosomal contents—including acidic hydrolases and ions—are released into the cytosol, leading to cytosolic acidification. Proteolytic enzymes, particularly cathepsins, subsequently degrade cytoskeletal components and organelles [6]. Moreover, LMP can initiate multiple forms of regulated cell death, including apoptosis, pyroptosis, ferroptosis, and necroptosis [7,8]. In the context of pathogen infection, the specific cell death modality induced by LMP depends on multiple factors, such as the type of pathogen, the extent of lysosomal damage, the host cell type, and the nature of the immune response. The interplay among these factors ultimately determines the local pathological processes and the overall outcome of infection. In this review, we focus on recent advances in the field, systematically summarizing the determinants of lysosomal membrane stability, methods for detecting LMP, and the modes of cell death mediated by LMP, as well as highlighting the roles of LMP in pathogen infection.

## 2. Methods for Detecting Lysosomal Membrane Permeabilization

**Figure 1:**
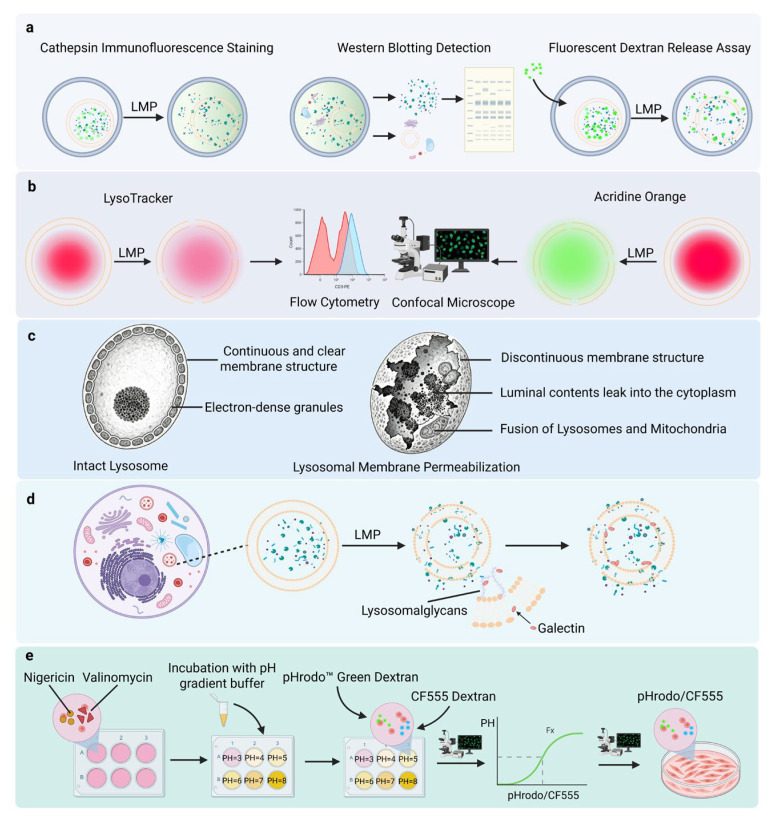
Methods for Detecting LMP. (**a**) Assessment of LMP by detecting the release of lysosomal contents into the cytosol. (**b**) Monitoring changes in lysosomal acidification and integrity using lysosome-targeting fluorescent probes. (**c**) Observation of ultrastructural features of lysosomal membranes by transmission electron microscopy. (**d**) Identification and discrimination of damaged lysosomal subpopulations based on the recruitment of galectins to compromised lysosomal membranes. (**e**) Indirect quantification of lysosomal membrane permeability using fluorescence-based methods calibrated by pH gradients.

The defining feature of LMP is the release of lysosomal contents into the cytosol. Accordingly, most detection strategies assess changes in lysosomal membrane permeability by monitoring the aberrant localization of lysosomal enzymes or tracer molecules (Figure 1). Immunofluorescence microscopy and differential centrifugation followed by Western blotting can reveal lysosomal enzyme release at the morphological and biochemical levels, respectively; however, their quantitative capacity is limited and they are susceptible to confounding factors such as antibody specificity and subcellular fractionation purity. Fluorescent dextran release assays, which evaluate the leakage of probes with different molecular weights, provide a functional assessment of LMP severity and pore size, but the results may be influenced by variability in endocytic uptake efficiency among cells [9]. Lysosome-targeting dyes are compatible with live-cell imaging and high-throughput screening, yet their signals primarily reflect lysosomal acidification rather than membrane integrity, making it difficult to accurately quantify the extent of LMP. Transmission electron microscopy offers direct ultrastructural evidence of lysosomal damage; however, its low throughput and interpretation-dependent nature limit its routine application. In contrast, galectin puncta assays enable the early discrimination between damaged and intact lysosomal subpopulations and are particularly useful for identifying partial or sublethal LMP. Quantitative approaches based on pH calibration can more precisely capture changes in lysosomal membrane permeability, but these methods are technically demanding and may introduce additional experimental perturbations [10,11]. Overall, no single method is currently sufficient to comprehensively characterize the dynamics and heterogeneity of LMP induced during pathogen infection. The combined use of multiple complementary techniques therefore remains a critical strategy for elucidating the mechanisms and biological consequences of LMP.

## 3. Damage Response Network to Lysosomal Membrane Permeabilization

**Figure 2:**
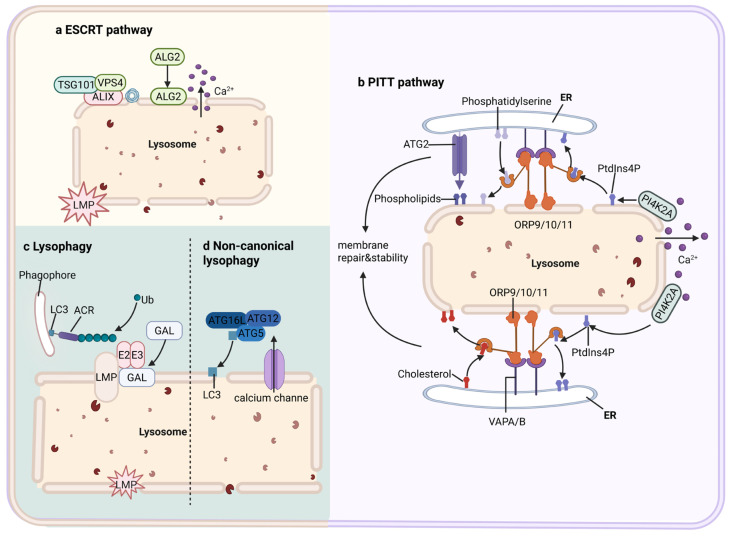
Repair Mechanism of Lysosomal Membrane. (**a**) When lysosomes are damaged, calcium release attracts repair proteins (ALG2, ALIX, TSG101, ESCRT-III, and VPS4) that seal breaks and reshape the membrane. This process creates internal vesicles that remove damaged areas. (**b**) Damage triggers PI4K2A recruitment, producing PtdIns4P. This recruits OSBP/ORP proteins that work with ER proteins (VAPA/VAPB) to transfer phosphatidylserine and cholesterol from ER to lysosomes. Phosphatidylserine then activates ATG2 to deliver lipids for membrane repair. (**c**) If damage is severe or irreparable, damaged lysosomes are eliminated. Exposed glycans recruit GALs and ubiquitin enzymes that tag lysosomal proteins. Autophagy receptors (ACRs) recognize these tags and recruit LC3 to initiate degradation. (**d**) LC3 directly couples to damaged lysosomal membranes, allowing fusion with healthy lysosomes for clearance.

Cells have evolved a complex and hierarchical damage response network to cope with LMP, which is composed of four interrelated branches. When lysosomes undergo only mild proton permeability defects without leakage of large luminal macromolecules, cells preferentially activate local membrane repair mechanisms to prevent damage from escalating into irreversible, complete LMP. In this context, the Ca^2+^-dependent endosomal sorting complex required for transport (ESCRT) pathway is rapidly engaged as an early response mechanism [12]. Through apoptosis-linked gene 2 (ALG-2) and ALG-2–interacting protein X (ALIX), ESCRT-III is recruited and assembled at damaged membrane sites to mediate rapid sealing of membrane lesions, typically within tens of seconds to one minute after injury [13]. In parallel, an ESCRT-independent phosphoinositide-initiated membrane tethering and lipid transport (PITT) pathway can operate at lysosome–endoplasmic reticulum membrane contact sites to facilitate lipid replenishment, serving as a context-dependent complementary repair mechanism [14,15]. When the extent or duration of membrane damage exceeds the cellular repair capacity, damaged lysosomes are recognized as dysfunctional organelles and are selectively removed through galectin-mediated lysosomal autophagy (lysophagy) [16]. In addition, during noncanonical autophagy (noncanonical LC3 lipidation), microtubule-associated protein 1 light chain 3 (LC3) can be directly conjugated to lysosomal membranes and interact with transient receptor potential mucolipin 1 (TRPML1), thereby promoting Ca^2+^ efflux, activating transcription factor EB (TFEB), and inducing lysosomal exocytosis. This process enhances lysosomal clearance and renewal, limiting secondary cellular damage caused by lysosomal content leakage [17]. Notably, lysophagy-mediated damage clearance strictly depends on the presence of a residual pool of functional lysosomes within the cell (Figure 2). Concurrently, lysosomal damage activates the mechanistic target of rapamycin complex 1 (mTORC1)–TFEB signaling axis, driving the transcriptional upregulation of lysosomal and autophagy-related genes to promote de novo lysosome biogenesis and regeneration, thereby restoring lysosomal functional capacity [18]. When lysosomal injury is severe and a substantial fraction of lysosomes undergo complete LMP, selective release of lysosomal contents, such as cathepsins, triggers multiple regulated cell death pathways, including apoptosis, pyroptosis, ferroptosis, and necroptosis. This tiered damage response network is essential for maintaining lysosomal homeostasis and plays a critical role in protecting against diverse pathological conditions, including neurodegenerative diseases and infections.

The diversity of damage response mechanisms indicates that distinct forms of lysosomal injury (Table 1) can elicit context-specific repair programs. In acute lysosomal damage models, such as exposure to membrane-lytic compounds or permeabilizing swelling agents, injury typically targets mature lysosomes and rapidly compromises membrane integrity. In response to such rapid and severe insults, cells predominantly rely on ESCRT- and PITT-mediated membrane repair pathways to promptly seal membrane lesions and restrict the spread of permeability [19,20]. In contrast, chronic stress caused by long-term particle accumulation or lysosomal storage disorders progressively weakens membrane stability, making cells more reliant on lysosomal renewal and metabolic remodeling to maintain homeostasis [21]. Pathogen infection represents a more complex scenario in which, alongside canonical repair mechanisms, immune-related membrane remodeling is further activated to limit membrane disruption and pathogen escape [22]. Overall, cells are capable of selectively engaging distinct repair and adaptive pathways according to the nature of lysosomal damage. Future studies are urgently needed to define the hierarchy and preferential deployment of individual repair mechanisms in response to specific types of lysosomal injury, as well as to dissect the crosstalk and regulatory networks linking these pathways. Such efforts will be essential for achieving a comprehensive understanding of the molecular basis by which lysosomes maintain structural and functional homeostasis under diverse stress conditions.

**Table 1:** Lysosomal membrane permeabilization inducers.

Category	Examples	Mechanism of Action	Refs.
Pathogens	*Listeria monocytogenes*, *Mycobacterium tuberculosis*, *Plasmodium* spp.	Induce excessive ROS generation, disrupt lysosomal ion/osmotic homeostasis, and secrete effector proteins or virulence factors to destabilize lysosomal membranes.	[23,24,25]
Alkalinizing compounds	Chloroquine, hydroxychloroquine, methotrexate	Protonation and lysosomal accumulation neutralize H^+^ and disrupt acidic homeostasis	[26,27]
Membrane-inserting agents	LLOMe, MSDH, GPN	Activated by lysosomal hydrolases to generate hydrophobic products/polymers that insert into lysosomal membranes, causing membrane destabilization.	[28,29,30]
Ionophores	Nigericin	Disrupt lysosomal ion balance, leading to pH imbalance, swelling, and membrane instability.	[31]
Exogenous particles	Asbestos fibers, silica crystals, nanoparticles	Trigger LMP via mechanical membrane destabilization and/or interactions with membrane lipids.	[32]
Metabolic dysregulation	Iron metabolism disorders; lipid metabolism disorders	Iron overload generates abundant •OH that oxidize lysosomal membrane lipids; lipid droplet accumulation and increased membrane rigidity compromise membrane stability.	[33,34]
Genetic defects	Lysosomal storage disorders; defects in autophagy-related genes	Deficiency of specific hydrolases causes substrate accumulation in lysosomes; impaired autophagosome–lysosome fusion leads to lysosomal functional overload.	[35,36]

L-leucyl-L-leucine methyl ester (LLOMe), O-methyl-serine-dodecylamide hydrochloride (MSDH), glycyl-L-phenylalanine 2-naphthylamide (GPN).

**Figure 3:**
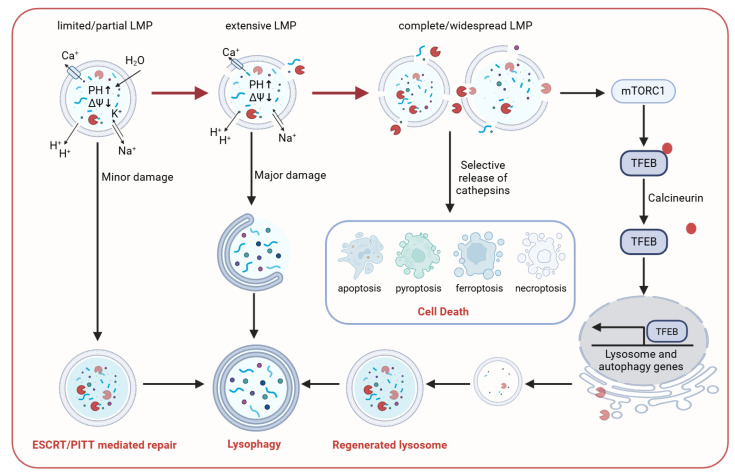
Damage Response Network to Lysosomal Membrane Permeabilization. Cellular outcomes following LMP are determined by the extent of membrane damage. Limited, partial LMP can be rapidly repaired through Ca^2+^-dependent ESCRT/PITT pathways. When repair fails or damage becomes excessive, injured lysosomes are recognized and sequestered, and subsequently removed via selective autophagy. Meanwhile, an mTORC1-driven signaling cascade promotes the biogenesis and regeneration of lysosomal components, thereby replenishing the cellular functional lysosome pool [41]. In contrast, complete and widespread LMP leads to massive cytosolic release of lysosomal contents such as cathepsins, thereby activating multiple cell-death pathways.

Accumulating evidence indicates that the extent of LMP is a central determinant of cell fate. This extent is mainly shaped by two interrelated factors: (i) the number and subtype of lysosomes affected, and (ii) pore size, stability, and duration. Together, these parameters determine the amount, composition, and kinetics of lysosomal content release, thereby setting thresholds and identities of downstream signaling pathways [37]. Under most stress conditions, LMP does not occur synchronously across the entire lysosomal population. Only when a critical fraction of lysosomes undergoes LMP within a short window do massive content release and loss of functional lysosomes drive irreversible damage and trigger cell-death programs [38]. However, the precise proportion of lysosomes required to trigger cell death remains to be determined. Moreover, lysosomal subtypes differ in size, membrane composition, luminal pH, and hydrolase profiles; thus, the released effectors and engaged pathways vary across lysosomal populations [39]. Importantly, LMP is not a simple “all-or-none” event. It remains unclear whether release occurs through transient, reversible pores or uncontrolled membrane rupture. Nevertheless, multiple studies suggest that LMP may exhibit molecular sieving properties. In apoptosis-related models, lower–molecular weight FITC–dextrans (10–40 kDa) are released from lysosomes, whereas larger molecules (≥70 kDa) are retained [40]. These observations indicate that pore size and stability not only determine the severity of LMP but may also shape the mode and kinetics of death signaling by regulating the identity and sequence of released lysosomal factors. This framework (Figure 3) centers on the extent of LMP and establishes a testable conceptual model that organizes cellular responses to lysosomal damage into a hierarchical sequence of “repair–clearance–regeneration–death,” thereby enabling prediction of how cell fate shifts among repair, adaptation, and death under different stress conditions.

## 4. Lysosomal Membrane Permeabilization-Mediated Cell Death

As a common gateway to multiple forms of cell death, whether LMP leads to a specific death outcome remains unresolved. Based on available evidence, we propose that the death phenotypes induced by LMP are jointly determined by cell type, microenvironmental context, and the intensity and kinetics of the stress stimuli. Different cell types exhibit intrinsic differences in lysosomal abundance, membrane composition, and luminal contents. In parallel, variations in intracellular signaling networks and molecular expression profiles further confer cell-specific “death preferences.” For example, macrophages express high levels of NLRP3 inflammasome components, enabling LMP to efficiently activate inflammasomes and preferentially trigger inflammatory cell death characterized by pyroptosis [42]. Notably, the molecular sieving properties and release kinetics of LMP also contribute to death modality selection. Studies using L-leucyl-L-leucine methyl ester (LLOMe)-induced LMP have demonstrated that the extent of lysosomal damage is not linearly correlated with NLRP3 inflammasome activity: low-dose LLOMe induces slow, partial LMP that favors inflammasome activation, whereas high-dose LLOMe causes rapid and widespread lysosomal collapse, paradoxically suppressing ASC oligomerization and IL-1β release [43].

In LMP-mediated apoptosis, lysosomes act as an upstream regulatory node of the mitochondrial apoptotic pathway. Cathepsins released upon LMP can cleave Bid into tBid, which subsequently activates Bax/Bak to induce mitochondrial outer membrane permeabilization (MOMP) and cytochrome c release, thereby triggering the caspase cascade and driving apoptosis [44]. In this process, the Bcl-2 family exerts coordinated cross-organelle regulation: Bcl-2/Bcl-xL suppress LMP, whereas Bax/Bak promote LMP [45]. In contrast to apoptosis, LMP in pyroptosis does not function as a direct execution event but rather serves as a permissive signal required for inflammasome activation. Lysosomal damage promotes NLRP3 inflammasome assembly, leading to the maturation and release of IL-1β and IL-18 and the formation of plasma membrane pores via gasdermin D (GSDMD) cleavage, culminating in cell lysis [46]. This process likely involves multiple proteases and disruptions in ion homeostasis, rather than relying solely on cathepsin B [47]. In ferroptosis, LMP represents a critical amplification node for metabolic death signals. Increasing evidence suggests that lysosomes may serve as initiation platforms for ferroptosis, wherein lipid peroxidation first induces LMP, followed by the release of large amounts of iron ions and reactive radicals. This process exacerbates lipid peroxidation and ultimately compromises plasma membrane integrity, driving ferroptotic cell death [34]. Cells with higher iron burden are therefore more prone to entering the ferroptotic program via this LMP-dependent amplification loop [33,48]. In necroptosis, LMP has recently been recognized as a noncanonical yet functionally important execution platform. Contrary to the traditional view that mixed lineage kinase domain-like protein (MLKL) primarily targets the plasma membrane or mitochondria, recent studies have shown that activated MLKL can translocate to lysosomal membranes, oligomerize, and directly induce LMP [49].

**Figure 4:**
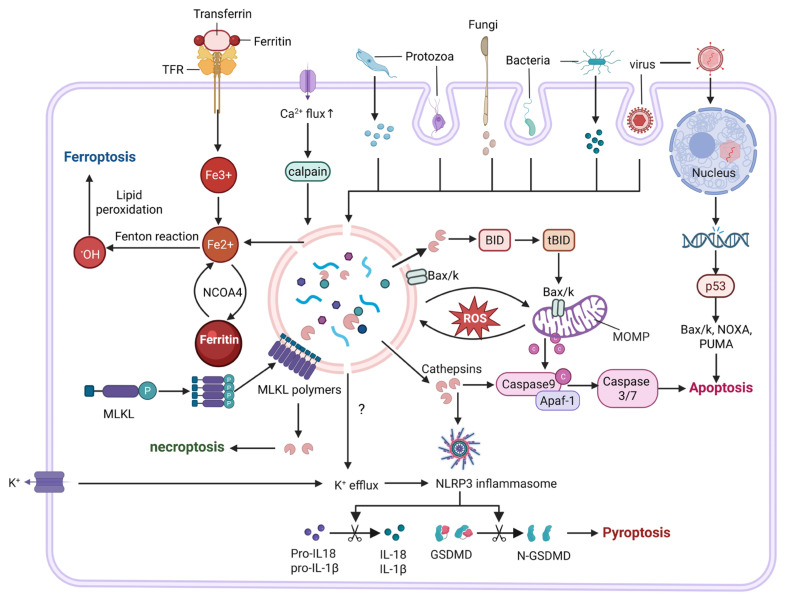
Lysosomes in cell death pathways. Pathogen infection and other stimuli can induce lysosomal damage through multiple mechanisms, leading to LMP and the release of lysosomal contents into the cytosol. Partial or mild LMP allows the translocation of cathepsins into the cytosol, thereby mediating lysosome-dependent apoptosis or, in immune cells, triggering inflammasome activation and pyroptosis. In other contexts, LMP-associated iron release and amplification of oxidative stress promote lipid peroxidation, ultimately leading to ferroptosis. In addition, extensive or catastrophic lysosomal rupture results in the uncontrolled release of cathepsins and other hydrolases, causing cytosolic acidification and culminating in necrotic or necroptosis-like cell death.

Collectively, pathogen-induced LMP constitutes a convergent node linking apoptosis, pyroptosis, ferroptosis, and necroptosis (Figure 4). These cell death programs do not operate as isolated pathways. For instance, when apoptosis is suppressed by infection, pharmacological intervention, or genetic mutations, necroptosis can function as a compensatory mechanism [50]. Moreover, induction of the major ferroptosis regulator glutathione peroxidase 4 (GPX4) has been shown to attenuate both ferroptosis and pyroptosis during bacterial infection [51]. Conversely, in human triple-negative breast cancer cells, glutathione (GSH) depletion has been reported to enhance necroptosis and ferroptosis, suggesting the existence of a potential signaling axis involving pyroptosis and necroptosis [52]. Together, these findings underscore that cell death pathways are not independent entities operating in parallel, but rather form an interconnected and dynamically regulated network.

## 5. Mechanisms and Consequences of Pathogen-Induced Lysosomal Membrane Permeabilization

Many pathogenic microorganisms need to enter intracellular compartments of host cells, or even the cytosol, to survive, replicate, and evade immune clearance [53]. Accumulating evidence indicates that a wide range of intracellular pathogens—including bacteria, viruses, protozoa, and fungi—can directly or indirectly induce LMP (Table 2), resulting in the leakage of lysosomal hydrolases, ions, and other bioactive molecules into the cytosol. This process can further inhibit autophagy, delay phagolysosome maturation, promote phagolysosomal escape, and even drive host cell death. Overall, the mechanisms by which pathogens induce LMP can be broadly classified into several categories.

Many pathogens directly compromise lysosomal membrane integrity by secreting pore-forming toxins (PFTs), thereby inducing LMP. Listeriolysin O (LLO), a cholesterol-dependent cytolysin secreted by *Listeria monocytogenes*, is selectively activated in acidic environments such as phagolysosomes (pH ≈ 5.5), where it forms membrane pores [54]. Leveraging this property, CD47–LLO antibody–toxin conjugates have been developed to enhance tumor antigen cross-presentation and cytosolic immune sensing by promoting phagocytosis and lysosome-confined release of LLO [55].

Pathogen infection or exposure to exogenous toxins can also markedly elevate intracellular reactive oxygen species (ROS) levels by inducing mitochondrial dysfunction or activating NADPH oxidases (NOX) [56]. Excessive ROS oxidize lysosomal membrane lipids and weaken membrane stability, thereby increasing lysosomal permeability. In Salmonella infection, for example, the type III secretion system 1 (T3SS-1)–translocated effector proteins SopE and SopE2 activate Rac1, promoting NOX2-dependent ROS production [57], while the phospholipase activity of SopB further exacerbates membrane stress [58]. Together, these effects increase the likelihood of LMP. It should be noted that ROS typically function as indirect amplifiers rather than direct triggers of LMP; during cell death processes such as ferroptosis and apoptosis, ROS accumulation can synergize with other stress signals to induce or exacerbate LMP, thereby amplifying death signaling [59].

Pathogens can also perturb lysosomal function by disrupting ionic homeostasis and osmotic balance, which may ultimately induce LMP. A representative example is the SARS-CoV-2 accessory protein open reading frame 3a (ORF3a) [60]. Mechanistically, pathogen-induced lysosomal damage appears to follow a dose–threshold model rather than a binary “LMP versus no LMP” outcome. Under low-intensity or transient exposure, pathogen-derived factors primarily disturb lysosomal ion homeostasis and induce deacidification, resulting in functional impairment without overt membrane rupture; such changes are often repairable or tolerable by the host cell. As pathogen burden increases, virulence factor expression intensifies, or exposure is prolonged, lysosomal stress progressively accumulates. Once this stress exceeds the cellular repair and buffering capacity, damage transitions from reversible functional dysfunction to irreversible structural disruption—namely, LMP—thereby triggering protease release, inflammatory amplification, and activation of cell death programs.

Beyond these general mechanisms, several specialized strategies have been identified. Viruses and bacteria that have coevolved with their hosts can exploit host cellular machinery to facilitate their propagation, including the induction of LMP-mediated cell death. For instance, in human immunodeficiency virus (HIV)-infected CD4^+^ T cells, upregulation of DNA damage-regulated autophagy modulator 1 (DRAM1) promotes LMP-mediated cell death, thereby facilitating viral dissemination [61]. In addition, certain fungi and parasites can induce LMP through direct physical damage. In malaria infection, for example, hemozoin crystals produced by *Plasmodium* spp. accumulate within macrophage phagolysosomes, where their sharp crystalline edges mechanically puncture lysosomal membranes and induce LMP [25].

In multicellular organisms, the elimination of infected cells to maintain tissue integrity is a fundamental principle of immune defense and critically shapes homeostasis and disease outcomes [62]. Pathogen infection often induces multiple forms of cell death across different cell types, and cell death is increasingly viewed as a double-edged sword during viral infection. Moderate levels of cell death constitute protective innate immune responses by directly limiting pathogen spread through elimination of infected cellular reservoirs. For example, LMP-induced pyroptosis leads to robust release of proinflammatory cytokines such as IL-1β and IL-18, enhancing vascular permeability and immune cell infiltration to promote efficient clearance of extracellular pathogens [63]. When LMP preferentially drives apoptosis, cells form membrane-intact apoptotic bodies that are quietly removed by neighboring phagocytes, which is more conducive to maintaining local immune homeostasis [64]. Conversely, uncontrolled cell death can result in dysregulated immune responses, cytokine storms, tissue damage, and enhanced pathogen dissemination [65]. Finally, cell death triggered by pathogen-induced LMP does not occur in a random or synchronous manner; rather, it is jointly regulated by the stage of infection, cell type, and the integrity of relevant signaling pathways, exhibiting a dynamic and temporally coordinated pattern with substantial plasticity. Moreover, extensive crosstalk exists among distinct cell-death pathways, such that when one pathway is inhibited, alternative death mechanisms can be engaged as compensatory routes.

**Table 2:** Mechanisms of LMP Induction by Different Pathogens.

Type	Pathogen	Mechanisms of LMP Induction	Refs.
Bacteria	*Listeria monocytogenes*	Secretes LLO, a pH-dependent pore-forming toxin that forms pores in phagolysosomal membranes.	[23,66]
	*Salmonellautilizes*	SopE and SopE2 activate NOX2-dependent ROS production and lipid peroxidation, while SopB disrupts membrane dynamics and homeostasis of the lysosomal system; together, these effectors cooperatively promote LMP.	[57,58]
	Uropathogenic *Escherichia coli*	HlyA forms pores in the lysosomal membrane, directly disrupting its physical integrity.	[67]
	*Mycobacterium tuberculosis*	By activating the type I interferon–ACOD1 axis, ACOD1 is induced to mediate the proteasomal degradation of the lysosomal stabilizing protein HSP70 in a non-catalytic manner in the cytosol, thereby inducing LMP.	[24]
Viruses	Human immunodeficiency virus	In HIV-infected CD4^+^ T cells, the expression of DRAM (DNA damage-regulated autophagy modulator 1) is upregulated, leading to the induction of LMP in infected cells.	[61]
	SARS-CoV-2	ORF3a-mediated lysosomal deacidification and disruption of ion and osmotic homeostasis.	[68]
Fungi	*Candida albicans*	Secreted candidalysin induces pore formation in phagolysosomal membranes, whereas hyphal extension provides an additional, mechanical mode of phagolysosomal disruption.	[69,70]
	*Aspergillus fumigatus*	Melanin regulates pH; secretes gliotoxin to induce oxidative stress and binds to sulfhydryl groups of lysosomal membrane proteins, compromising membrane stability	[71]
Protozoa	*Trypanosoma cruzi*	Trans-sialidase activated in acidic environment removes sialic acid from LAMP proteins; low pH-dependent pore-forming protein disrupts phagolysosomal membranes	[72]
	*Plasmodium* spp.	Hemozoin crystals cause physical puncture damage to lysosomal membranes	[25]

## 6. Conclusions and Prospects

Lysosomes serve dual roles as degradative organelles and as central platforms for host innate immunity, and a dynamic balance exists between pathogen-induced LMP and host defense responses. Despite the critical role of LMP in host–pathogen interactions, many fundamental questions remain unresolved. First, the dynamic progression of LMP is not fully understood: the spatiotemporal transition from localized membrane injury to complete membrane rupture, the differential effects of varying degrees of membrane permeabilization on cell fate, and the kinetics of post-damage membrane repair all lack systematic characterization. Second, the regulatory thresholds governing LMP have not been quantitatively defined. Precise quantification is needed to determine the extent of membrane permeabilization required to trigger antimicrobial responses versus cell death, to establish dose–response relationships for LMP induced by different pathogens, and to delineate cell type-specific sensitivities to LMP. In addition, the crosstalk between LMP and complex immune signaling networks remains incompletely understood; how LMP cooperates with or antagonizes autophagy pathways in pathogen clearance, how membrane damage activates distinct cell-death programs, and how the temporal hierarchy and prioritization of these signaling pathways are organized all warrant further investigation. Elucidating these issues will provide a comprehensive understanding of the regulatory networks governing LMP in infection immunity.

From a translational medicine perspective, therapeutic strategies targeting LMP show considerable promise for disease prevention and treatment. By modulating lysosomal membrane stability, it may be possible to achieve a precise balance between enhancing host antimicrobial responses and limiting excessive inflammatory damage [73]. Distinct therapeutic approaches can be tailored according to pathogen-specific patterns of LMP regulation: for pathogens that depend on an intact lysosomal environment, promoting LMP may enhance pathogen clearance; conversely, for pathogens that actively induce LMP, stabilizing lysosomal membranes may prevent pathogen escape or pathogenicity [74]. In summary, a deeper understanding of LMP mechanisms will provide an important theoretical foundation for elucidating the principles of host–pathogen interactions and for developing novel anti-infective therapeutic strategies.

## Data Availability

No new data were created or analyzed in this study. Data sharing is not applicable to this article.
